# Evolutionary relationships of the Critically Endangered frog *Ericabatrachus baleensis* Largen, 1991 with notes on incorporating previously unsampled taxa into large-scale phylogenetic analyses

**DOI:** 10.1186/1471-2148-14-44

**Published:** 2014-03-10

**Authors:** Karen Siu-Ting, David J Gower, Davide Pisani, Roman Kassahun, Fikirte Gebresenbet, Michele Menegon, Abebe A Mengistu, Samy A Saber, Rafael de Sá, Mark Wilkinson, Simon P Loader

**Affiliations:** 1 Molecular Evolution and Bioinformatics Lab, National University of Ireland, Maynooth, Co. Kildare, Ireland; 2 School of Biological Sciences and School of Earth Sciences, Woodland Road, Bristol BS8 1UG, UK; 3 Department of Life Sciences, The Natural History Museum, Cromwell Road, London SW7 5BD, UK; 4 Ethiopian Wildlife Conservation Authority, P.O. Box 386, Addis Ababa, Ethiopia; 5 Department of Zoology, Oklahoma State University, 311 D Life Sciences West, Stillwater, OK 74078, USA; 6 Tropical Biodiversity section, MUSE - Museo delle Scienze di Trento, Viale del Lavoro e della Scienza 3, Trento 38123, Italy; 7 Department of Environmental Sciences, University of Basel, Biogeography Research Group, Basel 4056, Switzerland; 8 Zoology Department, Faculty of Science, Al-Azhar University, Assiut, Egypt; 9 Department of Biology, University of Richmond, Richmond, VA 23173, USA

**Keywords:** Africa, Amphibia, Eastern Afromontane, Ethiopia, *Petropedetes*, phylogenetics

## Abstract

**Background:**

The phylogenetic relationships of many taxa remain poorly known because of a lack of appropriate data and/or analyses. Despite substantial recent advances, amphibian phylogeny remains poorly resolved in many instances. The phylogenetic relationships of the Ethiopian endemic monotypic genus *Ericabatrachus* has been addressed thus far only with phenotypic data and remains contentious.

**Results:**

We obtained fresh samples of the now rare and Critically Endangered *Ericabatrachus baleensis* and generated DNA sequences for two mitochondrial and four nuclear genes. Analyses of these new data using *de novo* and constrained-tree phylogenetic reconstructions strongly support a close relationship between *Ericabatrachus* and *Petropedetes*, and allow us to reject previously proposed alternative hypotheses of a close relationship with cacosternines or *Phrynobatrachus*.

**Conclusions:**

We discuss the implications of our results for the taxonomy, biogeography and conservation of *E. baleensis*, and suggest a two-tiered approach to the inclusion and analyses of new data in order to assess the phylogenetic relationships of previously unsampled taxa. Such approaches will be important in the future given the increasing availability of relevant mega-alignments and potential framework phylogenies.

## Background

*Ericabatrachus baleensis* Largen, 1991, the sole member of its genus, is poorly known and Critically Endangered frog known only from the Harenna Forest in the Bale Mountains of Ethiopia and, until recently, only from the original collection made in 1986 [1-4]. In his description of the genus and species, Largen [5] noted that he intended but had not yet managed to study comparative osteology, which might have provided compelling insights into the evolutionary affinities of *Ericabatrachus*. Instead, while noting that *Ericabatrachus* was “reminiscent of *Phrynobatrachus*” (p. 147) with “habitus *Phrynobatrachus*-like” (p. 141), Largen tentatively concluded, on the basis of shared external features such as terminally T-shaped (“bifid”) phalanges, that *Ericabatrachus* is a petropedetine (= petropedetid of some classifications). Thus, in this view, *Ericabatrachus* is most closely related to the East African *Arthroleptides* Nieden, 1911 (= *Petropedetes* Reichenow, 1874), East and West African *Petropedetes* Reichenow, 1874, and Central/West African *Phrynodon*, Parker, 1935. Petropedetinae/dae is a putatively monophyletic group nested within the large clade of “True Frogs” [6] termed “ranids” [7,8].

**Figure 1:**
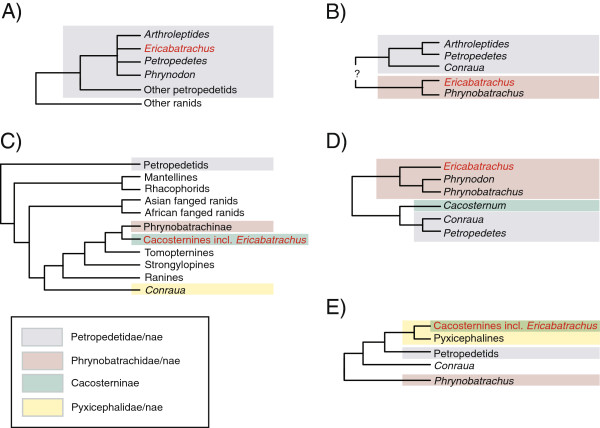
**Alternative hypotheses of the relationships of *Ericabatrachus baleensis *and its sister groups.** The hypotheses are derived from different sources, which were at the time not necessarily presented as trees (see also Table 1). **A)** Largen [5], **B)** Dubois [8], **C)** Scott [9] (based on her Figure 4, the consensus of morphological and molecular analyses and the revised classification in Appendix seven), **D)** Frost et al. [6], and **E)** Pyron and Wiens [10].

Uncertainty over the affinities of *Ericabatrachus* is reflected in a period of taxonomic instability from 2005 until present (summary in Table 1 and Figure 1). Dubois [8] suggested an affiliation between *Ericabatrachus* and *Phrynobatrachus*, presumably based on the similar habitus and superficial resemblance noted by Largen [5]. The same year, Scott [9] published the first broad-scale analysis of ranid phylogeny based on both morphology (predominantly osteology, including the first data for *Ericabatrachus*) and DNA sequence data (lacking for *Ericabatrachus*). Scott’s [9] analyses recovered *Ericabatrachus* within the primarily southern African cacosternids, separate from phrynobatrachines and only distantly related to petropedetines (Figure 1). Subsequently, substantial changes to amphibian classification were proposed [6,10] on the basis of large-scale phylogenetic analyses of mostly or entirely DNA sequence data, respectively. Neither of these studies included *Ericabatrachus* in their phylogenetic analysis, but *Ericabatrachus* was alternatively classified within Phrynobatrachidae, considered as likely nesting within *Phrynobatrachus* based on Largen’s [5] comment that the taxon was “*Phrynobatrachus*-like” [6] or classified within Pyxicephalidae based on Scott’s [9] findings [10]. In summary, over the past 22 years *Ericabatrachus* has been treated as a member of three different families.

**Table 1:** **Chronological account of the taxonomic arrangement of *Ericabatrachus baleensis *** **Largen, 1991**

**Author, Year**	**Family**	**Subfamily**	**Genera included**
Frost, 1985	**Petropedetidae Noble, 1931**		*Anhydrophryne* Hewitt, 1919;
			*Arthroleptella* Hewitt, 1926;
			*Arthroleptides* Nieden, 1910;
			*Cacosternum* Boulenger, 1887;
			*Dimorphognathus* Boulenger, 1906;
			*Microbatrachella* Hewitt, 1926;
			*Natalobatrachus* Hewitt & Methuen, 1913;
			*Nothophryne* Poynton, 1963;
			*Petropedetes* Reichenow, 1874
			*Phrynobatrachus* Günther, 1862;
			*Phrynodon* Parker, 1935.
Largen, 1991	**Petropedetidae Noble, 1931**		*Anhydrophryne* Hewitt, 1919;
			*Arthroleptella* Hewitt, 1926;
			*Arthroleptides*, Nieden, 1910;
			*Cacosternum* Boulenger, 1887;
			*Dimorphognathus* Boulenger, 1906;
			***Ericabatrachus *** **Largen, 1991;**
			*Microbatrachella* Hewitt, 1926;
			*Natalobatrachus* Hewitt & Methuen, 1913;
			*Nothophryne* Poynton, 1963;
			*Petropedetes* Reichenow, 1874;
			*Phrynodon* Parker, 1935;
			*Poyntonia* Channing & Boycott, 1989.
Dubois, 2005	Petropedetidae Noble, 1931		*Arthroleptides* Nieden, 1910;
			*Conraua* Nieden, 1908;
			*Petropedetes* Reichenow, 1874;
	**Phrynobatrachidae Laurent, 1941**		*Phrynobatrachus* Günther, 1862;
			***Ericabatrachus *** **Largen, 1991.**
Scott, 2005	Ranidae Rafinesque-Schmaltz, 1814	Phrynobatrachinae Laurent, 1941	*Natalobatrachus* Hewitt & Methuen, 1913;
			*Phrynobatrachus* Günther, 1862;
		Petropedetinae Noble, 1931	
			*Petropedetes* Reichenow, 1874; > (synonymized *Arthroleptides* Nieden, 1910);
		**Cacosterninae Noble, 1931**	*Anhydrophryne* Hewitt, 1919;
			*Arthroleptella* Hewitt, 1926;
			*Cacosternum* Boulenger, 1887;
			***Ericabatrachus *Largen, 1991**;
			*Microbatrachella* Hewitt, 1926;
			*Nothophryne* Poynton, 1963;
			*Poyntonia* Channing & Boycott, 1989.
Frost, 2006	Petropedetidae Noble, 1931		*Conraua* Nieden, 1908;
			*Petropedetes* Reichenow, 1874;
			Indirana Laurent, 1986;
	Phrynobatrachidae Laurent, 1941		***Ericabatrachus *Largen, 1991**;
			*Phrynobatrachus* Günther, 1862;
			(*Phrynodon* Parker, 1935) * placed in synonymy of *Phrynobatrachus*.
Roelants et al., 2007	Petropedetidae Noble, 1931		*Conraua* Nieden, 1908;
			*Petropedetes* Reichenow, 1874; > (removal of *Indirana* Laurent, 1986).
Pyron and Wiens, 2011	Petropedetidae Noble, 1931		*Petropedetes* Reichenow, 1874;
	Phrynobatrachidae Laurent, 1941		*Phrynobatrachus* Günther, 1862;
	**Pyxicephalidae Bonaparte, 1850**	**Cacosterninae**	*Amietia* Dubois, 1987;
			*Anhydrophryne* Hewitt, 1919;
			*Arthroleptella* Hewitt, 1926;
			*Cacosternum* Boulenger, 1887;
			***Ericabatrachus *Largen, 1991**;
			*Microbatrachella* Hewitt, 1926;
			*Natalobatrachus* Hewitt & Methuen, 1913;
			*Nothophryne* Poynton, 1963;
			Poyntonia Channing & Boycott, 1989;
			*Strongylopus* Tschudi, 1838;
			*Tomopterna* Duméril and Bibron, 1841;
		Pyxicephalinae	*Aubria* Boulenger, 1917;
			*Pyxicephalus* Tschudi, 1838;
	Conrauidae		*Conraua* Nieden, 1908.

Text in bold indicates placement of *E. baleensis*.

With newly collected specimens of *Ericabatrachus baleensis* (see [4,11]), DNA sequence data can, for the first time, be used to investigate the phylogenetic relationships of this challenging taxon. Inferring the phylogenetic relationships of *Ericabatrachus* has important implications for both its biogeography and conservation. If *Ericabatrachus* is closely related to Petropedetidae, this would support the Afromontane biogeographic region [12]. Alternatively, relationships shared with predominantly southern African taxa (either Pyxicephalidae or Phrynobatrachidae) would provide evidence of an unusual biogeographical association. Phylogeny is an important consideration in conservation prioritization (e.g. [13]) and resolution of the relationships of *Ericabatrachus* will shed light on the validity of *Ericabatrachus* as a monotypic genus and the degree to which this now Critically Endangered frog [14] contributes to the genetic distinctiveness of conservation targets in the generally threatened [4] Bale Mountains of Ethiopia.

Substantial steps have recently been made in resolving amphibian phylogenetic relationships [6,10]. The existence of a large and relatively well–sampled mega-alignment including more than 2,800 amphibians [10], potentially provides a useful basis for investigating the phylogenetic position of previously unsampled taxa [15] such as *Ericabatrachus*. However, what might constitute best use of prior phylogenetic work and resources is not necessarily obvious. For example, should we simply append or shoehorn data for new taxa into an existing mega-alignment, thereby accepting previous strategies employed in marker selection, alignments, and masking or should we re-evaluate some or all of these? Should we accept previous phylogenetic conclusions and use these as topological constraints in order to expedite efficient placement of the newly included taxa or should we begin time-consuming unconstrained analyses *de novo*? Here we use newly generated DNA sequence data to investigate the phylogenetic relationships of *Ericabatrachus* and some of the possible strategies for incorporating previously unsampled taxa into large-scale phylogenetic analyses.

## Results

### Saturation analysis

Saturation plots (reported in Additional file 1) supported the inclusion of the following partitions in the large-scale phylogenetic analysis: *RAG1* codon positions 1, 2 and 3; *H3A* codon positions 1 and 2; *16S*; *12S; 28S; CXCR4* codon positions 1, 2 and 3; *SLC8A1* codon positions 1, 2 and 3; *POMC* codon positions 1, 2 and 3; *RHOD* codon positions 1 and 2; *SIA* codon position 2; *SLC8A3* codon positions 1, 2 and 3; *TYR* codon positions1 and 2; and *cytb* codon positions 1 and 2. An outlier species was detected in the *28S* saturation plot, *Fejervarya limnocharis*, and this marker was excluded for this taxon from the analysis.

### Phylogenetic analyses

**Figure 2:**
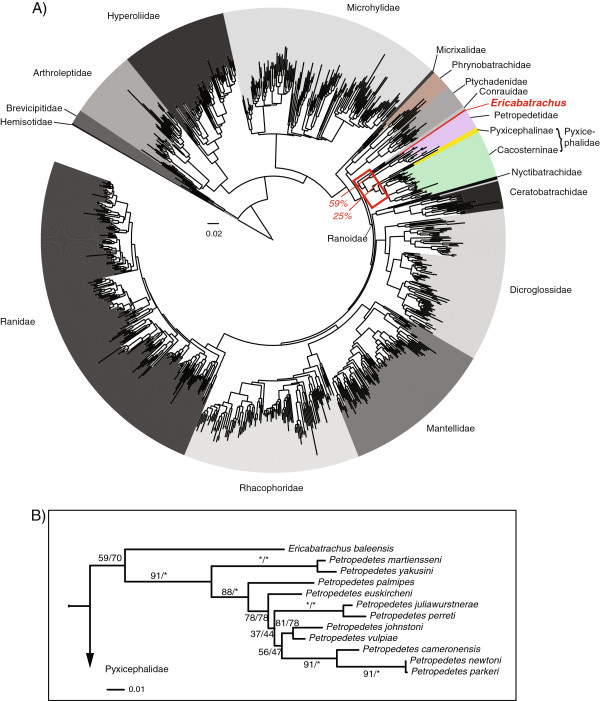
**Phylogenetic relationships of *Ericabatrachus baleensis*. A)** ML tree from the large-scale analysis of Ranoidea. Most frequent placements for *Ericabatrachus* with the corresponding bootstrap percentages are shown (red arrows). The red square denotes the area where *Ericabatrachus* joins the tree 99% of the times (see text). **B)** Close up view of position of *Ericabatrachus* as the sister of *Petropedetes*. Support values for each branch correspond to (left) the *de novo* analysis and (right) the constrained analysis.

The large-scale (858-taxon data set), unconstrained ML analysis recovered *Ericabatrachus* as the sister taxon of *Petropedetes* with a bootstrap support of 59% (Figure 2A). This low bootstrap support is primarily a consequence of *Ericabatrachus* being associated with other clades in 35% of the bootstrap replicates (BR) (Additional file 2) but is contributed to also by the instability of *Petropedetesnewtoni* which was found outside of *Petropedetes* + *Ericabatrachus* in 9% of the BR. Hence, the effective support for an *Ericabatrachus*-*Petropedetes* (with exclusion of *P. newtoni*) relationship is 65% (Additional file 2). The second most frequent position (25% of the BR) places *Ericabatrachus* as the sister to or nested inside Pyxicephalinae (the clade composed of *Aubria* + *Pyxicephalus*). Taken together these results circumscribe a relatively well-defined area of the tree within the Ranoidae (or Natatanura [6]) in Figure 2A, including the following lineages: Pyxicephalidae + Petropedetidae + Conrauidae, in which *Ericabatrachus* occurs with a cumulative bootstrap proportion of ~99% (Additional file 2). This allows narrowing the set of plausible relationships for *Ericabatrachus*, and permits more focused analyses to be performed. Using Pyron and Wiens' [10] tree as a topological constraint produced very similar results with respect to the position of *Ericabatrachus* and the added *Petropedetes* species, including similar bootstrap support scores (Figure 2B).

**Figure 3:**
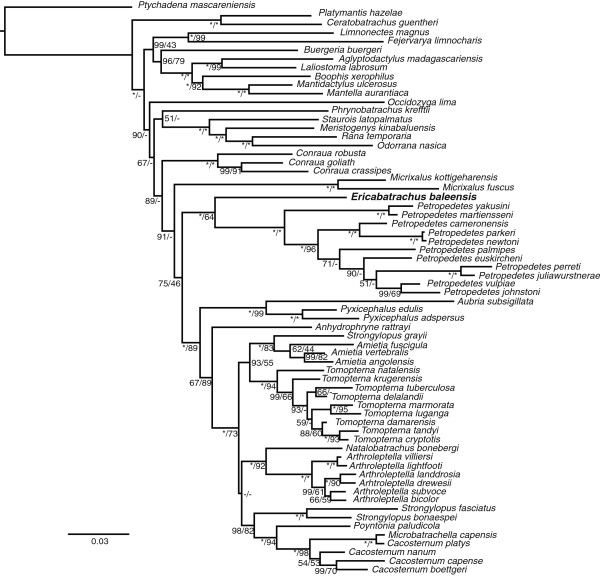
**Small-scale Bayesian tree under GTR model showing the phylogenetic placement of *Ericabatrachus baleensis *(in bold).** Support values for the nodes correspond to posterior probabilities (left) and non-parametric bootstraps (right). Values with “*” represent maximal support (100%), values lower than 40% are denoted by “-”.

Focused, smaller-scale Bayesian and ML analyses (66-taxon data set) recover *Ericabatrachus* as most likely the sister group to *Petropedetes* (Figure 3). The posterior probability for this position under the GTR + G, CAT + G, or CAT-GTR + G models is invariably equal to one. ML bootstrap support is only marginally increased (to ~ 67%). The topologies obtained in different analyses of the 66-taxon data set are almost identical, varying only in the positions of Occidoziga lima, *Phrynobatrachus kreffti*, and *Micrixalus*. AU tests show that the phylogenetic placement of *Ericabatrachus* obtained in our Bayesian and ML results fits the 66-taxon data significantly better than any previously proposed hypothesis.

**Figure 4:**
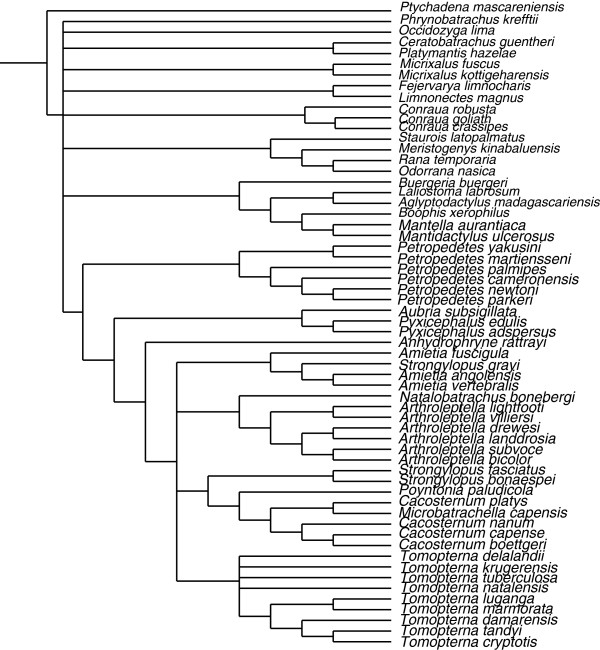
**Strict consensus of our small-scale Bayesian tree and Pyron and Wiens’ **[10]**tree.** Both trees were restricted to the common taxa. Polytomies represent relations that were in disagreement between the two trees.

The strict consensus of our small-scale Bayesian tree and the Pyron and Wiens’ [10] tree (both restricted to the common taxa) includes a large basal polytomy but is well resolved in the area where the new taxa (*Ericabatrachus* and some *Petropedetes* species) join the tree (Figure 4). There is a more substantial difference in log-likelihoods between these two trees with our alignment (24.2) than with the Pyron and Wiens’ [10] alignment (8.3), but results of AU tests of these restricted topologies using either our alignment or that of Pyron and Wiens [10] were not significant (p = 0.089 and p = 0.331 respectively).

## Discussion

### Taxonomy, phylogeny and biogeography

Thorough phylogenetic analyses of newly acquired molecular data for the rare and Critically Endangered *Ericabatrachus baleensis* provide good support for a sister-group relationship with *Petropedetes* Reichenow, 1874. Our results support Largen’s [5] original assignment of *Ericabatrachus* to the family Petropedetidae (although his concept of “Petropedetidae” was somewhat different from current taxonomy), rather than Scott’s ([9]: p. 532) conclusion that there is “no doubt that… *Ericabatrachus* is a cacosternine, not a petropedetine”. Largen suspected this petropedetid relationship on the basis of the presence of terminally T-shaped phalanges ([5,9]). Alternative groupings proposed more recently by other authors are not supported by our analyses. In the bootstrap replicates *Ericabatrachus* joins the tree only once at the base of Phrynobatrachidae (as proposed by Frost et al. [6]) and never in Cacosterninae (as proposed by Scott [9]). In terms of evolutionary relationships within “ranids”, in our analysis Petropedetidae forms a strongly supported sister group to a southern African radiation of ranids (Pyxicephalidae), with Conrauidae lying outside this pairing. Other possible resolutions are rejected by the AU tests (Table 2).

**Table 2:** Hypothesis-testing results

**Rank**	**Item**	**AU test**
1	present work, Bayesian GTR Tree	0.853
2	present work, ML Tree	0.262
**Hypotheses with AU test values lower than 0.05 are rejected**		
3	Dubois [8] hypothesis	0.008
4	Pyron and Wiens [10] hypothesis	1.00E-05
5	Frost et al. [10] hypothesis	1.00E-05
6	Scott [10] hypothesis	4.00E-08

Values shown for the Approximately Unbiased test (AU test) from CONSEL [35] tested with the 66-taxon data set (explained in detail in the Methods section). Dotted line separates the non-rejected hypotheses (above) from the rejected hypotheses (below).

The genus *Petropedetes sensu*[9] comprises 12 nominal species distributed in both East and Central Africa. Largen [5] was aware of the high degree of morphological dissimilarity between *Ericabatrachus* and other petropedetids (*Petropedetes*, *Arthroleptides* (=*Petropedetes*) and *Phrynodon* (=*Phrynonbatrachus sandersoni*)) and he was not drawn on any particular putative sister-group relationship. It might have been suspected that, given the geographical proximity of the highlands of Kenya and Tanzania, and the relative but fragmented biogeographic continuity of this area with the Ethiopian highlands, *Ericabatrachus* was most closely related to *Petropedetes* from East Africa (paralleling suspected relationships for other eastern African montane frogs such as in brevicipitids and bufonids [3,5,16,17]). An East African unit (*Ericabatrachus, P. martiennseni, P. yakusini*) is not supported in our phylogenetic analyses. Sampling of *Petropedetes* is almost complete, but data are lacking for *P. dutoiti* and *P. natator*the sister of Petropedates and the monophyly of *Petropedetes* awaits to be tested fully [18], and might alter our understanding of the relationship of *E. baleensis* relative to all known *Petropedetes*.

*Ericabatrachus* has been one of the most difficult genera of African ranids to classify. Efforts were hampered by the lack of molecular data, and uncertainty was compounded by the fact that *Ericabatrachus* has a suite of morphological characters that have seemingly confused understanding of its evolutionary relationships. Characters that might have supported Largen’s conclusion that *Ericabatrachus* was a petropedetid were seemingly not revealed in Scott’s [9] analysis. A re-assessment of the morphology of *Ericabatrachus* would clearly be interesting, particularly given the still incomplete knowledge of *Ericabatrachus*. Furthermore, as previously noted by Largen ([5]; p.151), *Ericabatrachus* would appear to be an interesting taxon to include in investigations of correlated patterns of evolution in geographically isolated localities in riverine adapted African ranid species.

On morphological grounds, *Ericabatrachus* seems to be highly divergent from many other close relatives (see Appendix four in [9] for list of characters), and this is further supported by molecular differences outlined in this study. A phylogenetic position outside of *Petropedetes*, the morphological distinctiveness of the taxon, and likely long period of divergence from its closest relatives (based on sequence differences for standard genetic markers) agree with Largen’s [5] original hypothesis that *Ericabatrachus* should be recognized as a distinct genus. Further research into the still rather complex, and fluctuating taxonomy of African ranids, will be necessary before a full and suitable nomenclatural resolution of Petropedetidae can be made [18].

Biogeographically *Ericabatrachus* has fascinated herpetologists since its original description. It is restricted to the high montane forest of the Bale Mountains, part of the fragmented chain of the Afromontane region [12]. Ethiopia is the most northerly, and therefore isolated part of an extensive chain of mountains in subSaharan Africa. In addition to *Ericabatrachus*, other monotypic amphibian endemics are known from Ethiopia and, along with other animal and plant groups, give rise to the impression that the region is a refuge for old and divergent taxa – often referred to as palaeoendemics. Based on branch lengths in our inferred phylogenies, we suspect that the divergence of *Ericabatrachus* from its closest extant relatives is very old given previous estimates of divergence times with closely related pairings in Petropedetidae, Pyxicephalidae and Conrauidae (e.g., [19,20]). The phylogenetic results reported here provide support for the idea that *E. baleensis* is a palaeoendemic species. In light of the other putative palaeoendemic taxa (e.g. *Balebreviceps* and *Altiphrynoides*), the Bale Mountains of Ethiopia appear to have an intriguing, ancient biogeographic history [17].

### Conservation

*Ericabatrachus baleensis* has declined substantially since its description, it has not been recorded at its type locality (Tulla Negesso) since 1986 or at the only other known historical locality (Katcha) since its original collection [4] and it has recently been re-assessed as Critically Endangered on the IUCN Red List [14]. The declines in these localities are likely to be in association with substantial human-induced habitat degradation in the Rira catchment area [4], but also possibly the emergent infectious disease amphibian chytridiomycosis [11]. We were able to locate *E. baleensis* only in Fute, a new locality in less degraded habitat than nearby Tulla Negesso. Our phylogenetic results demonstrate that the extinction of this frog would be a considerable loss of evolutionary history, thus adding to the demand [4] that urgent conservation action is taken. This could include both *ex situ* or *in situ* approaches, but given the co-occurrence of other distinctive, potentially palaeoendemic taxa in this locality – a more integrated *in situ* conservation action would be welcomed.

### Incorporating previously unsampled taxa into large-scale phylogenetic analyses

With the collection of previously unsampled taxa of quite uncertain phylogenetic relationships, such as *Ericabatrachus*, then (ignoring the choice of markers) one might try to find closely related taxa to include in a phylogenetic analysis with a BLAST search database query, produce an alignment, and analyse it as exhaustively as seems worthwhile. However, in the age of large-scale phylogeny projects, researchers are increasingly likely to have access to relevant mega-alignments and trees from previous phylogenetic studies. Such resources might greatly simplify and speed up the inference of phylogenetic relationships of previously unsampled taxa. For example, expanding the data through profile alignment and using previous trees as topological constraints can greatly reduce the computational complexity and expense of large-scale phylogenetic inference.

Of course, relying upon previous alignments and trees carries the risk that they are not optimal, particularly given that the inclusion of additional taxa (and markers) has the potential to change the inferred interrelationships of other taxa. In the absence of resource limitations (time, computer power, energy) we might consider *de novo* alignment and unconstrained phylogenetic analyses to be the optimal use of the new data because it would avoid such risks. But resources are always limited. Practical strategies must address the trade off between seeking to use previous results to speed up analysis (and avoid squandering resources) and seeking to avoid suboptimal inferences.

Here, our main strategy was to use the previous study of Pyron and Wiens [10] as a convenient source of aligned data and as a guide as to the taxonomic content of a major clade that background knowledge suggested included *Ericabatrachus*. We expanded Pyron and Wiens’ alignment with taxa and an additional marker and conducted *de novo* large-scale analyses that, in turn, informed taxon selection for subsequent smaller-scale analyses using additional methods and models. Different from [10], our *de novo* analyses included removal of seemingly saturated data partitions, which is generally considered to be helpful in phylogenetic analyses [21-24]. Substantial topological differences between Pyron and Wiens’ [10] and our tree (Figure 4) result from these differences in the data and its analyses. Although AU tests do not allow rejection of either tree, the topological differences highlight relationships that are probably best considered uncertain. In turn, this might be taken to suggest that the alternative strategy, of using the Pyron and Wiens [10] tree as a topological constraint, would be problematic. However, this is not the case in this instance. Both our *de novo* analyses and use of Pyron and Wiens’ [10] tree as a topological constraint recovered the same relationships of *Ericabatrachus*. We consider the agreement in this particular case to be a fortuitous consequence of the fact that incongruence between our and Pyron and Wiens’ [10] trees is concentrated in areas that are least relevant to the relationships of the previously unsampled *Ericabatrachus*.

Eventually it will be neither practical nor sensible to conduct large-scale *de novo* analyses each time a new sequence is added to an alignment. Thus, we anticipate that the use of topological constraints in phylogenetic analyses aimed at placing previously unsampled taxa will increase. We recommend use of topological constraints particularly where relationships have been recovered in multiple unconstrained analyses and appear to be well supported. Conversely we would advise against uncritical acceptance of previous topologies that are not well-corroborated.

When adding novel sequences for genes already present in an existing alignment, we recommend that the inclusion of new data is followed by either an analysis of saturation (as was carried out in this work) or a “quick and dirty” phylogenetic analysis for each gene partition to detect potential sequencing errors or contaminations. If adding entire new gene partitions, then we recommend conducting a BLAST search of the available sequences for that gene, followed by an analysis of saturation and a “quick and dirty” phylogenetic tree of each gene.

## Conclusions

The existence of relatively well-sampled large-scale alignments provided a potentially useful backbone to analyse the taxonomic placement of the poorly known Ethiopian frog *Ericabatrachus baleensis*. A two-tiered approach of phylogenetic analyses using ML and Bayesian methods showed that *Ericabatrachus* is the sister group of *Petropedetes*, which is supported by limited morphological evidence. All previous hypotheses of placement are statistically rejected based on our data set. Using a constrained tree yields the same phylogenetic position for *Ericabatrachus* demonstrating how this approach may obviate the need for time consuming *de novo* analyses. In general, constraints should be relied upon only when they are very well-supported. The sister-group relationship of *Ericabatrachus* and *Petropedetes* and the validity of *Ericabatrachus* as a separate and divergent genus support the contiguity of the Afromontane region and reinforces the importance of continuing conservation efforts in the Bale Mountains of Ethiopia.

## Methods

### Sampling and DNA extraction

Our survey of amphibians in Bale Mountains was given permission by federal and regional authorities in Ethiopia. Permission to collect and export material was facilitated by the Ethiopian Wildlife Conservation Authority. The project was part of a broader project to understanding Ethiopian amphibians in which a memorandum of understanding between University of Basel and Addis Ababa University was signed.

**Figure 5:**
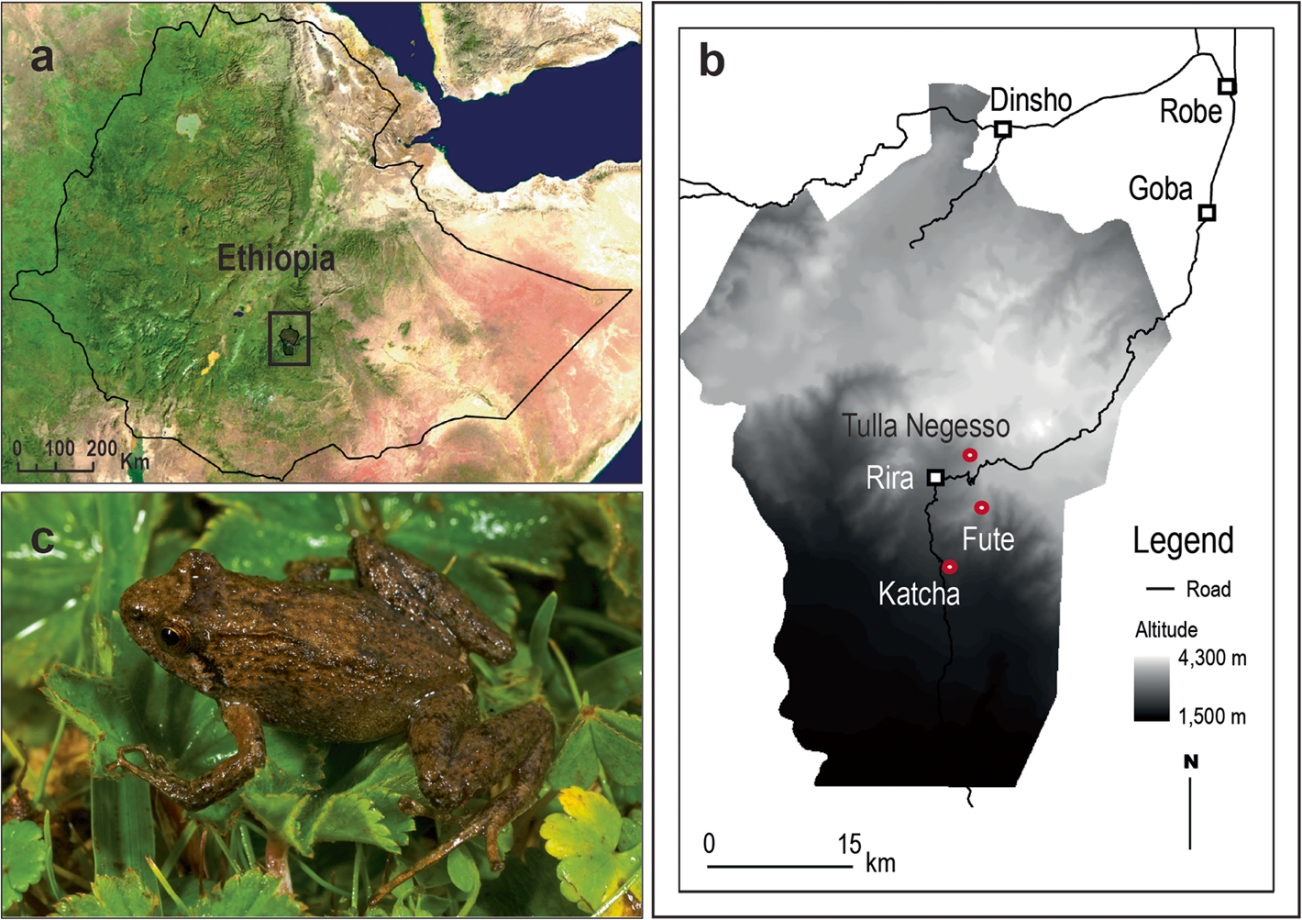
***Ericabatrachus baleensis *and its reported localities. A)** Map showing the Bale Mountains National Park in Ethiopia. **B)** Close-up of the Bale Mountains National Park showing the geographic position of the type locality (Tulla Negesso) and other sites (circles; squares indicate main human settlements). **C)** A specimen of *E. baleensis* found in the recent surveys [4], photograph by MM.

Fieldwork was conducted in July to August in 2008 in southeastern Ethiopia (Figures 5a–b) and June 2009, in Harenna Forest in Bale Mountains National Park. Harenna Forest is the type locality of *Ericabatrachus baleensis*[5], and comprises patchy, montane, primary rain forest, and secondary vegetation [4,5,11,25]. Herpetofaunal surveys carried out consisted primarily of visual encounter including rolling logs/stones and searching through leaf litter. All specimens for this study were collected in accordance with animal ethics guidelines established in the University of Basel.

In 2008, collected amphibian specimens, including a single sample of *Ericabatrachus baleensis* (ZNHM-AAU-A2013-003). The specimen was collected at a site in Harrena Forest called “Fute” (6.76474 N 39.751661 E, at 3208 m). Almost one year later (20th June 2009), a further two specimens (ZNHM-AAU-A2013-001, ZNHM-AAU-A2013-002) phenotypically similar to the first specimen and those of Largen [5], were secured at the same locality [4] (see Figure 5c). Specimens were anaesthetized using MS222, fixed in ca. 5% formalin, rinsed in water and stored in 70% ethanol in the collections of the Natural History Museum of Addis Ababa, Ethiopia. Tissue samples (liver) were taken from specimens prior to fixation and preserved in absolute ethanol.

Genomic DNA was extracted from each of the three *Ericabatrachus baleensis* liver samples with a Qiagen DNeasy kit using the protocol for purification of Total DNA from Animal Tissues. For the 2008 sample, we amplified and sequenced two partial mitochondrial genes, 12SrRNA (12S), and *16S*rRNA (*16S*) and three nuclear genes, 28SrRNA (28S), Histone *H3a* (*H3A*), and recombinase activating protein 1 (*RAG1*). In addition, we sequenced 12S, *16S* and *RAG1* for the 2009 samples to assess intraspecific variation (see Additional file 3 for details), where no major differences were found. Primers used in this study are given in Additional file 3.

### Data Matrix

To investigate the phylogenetic relationships of *Ericabatrachus* we added the newly sequenced data to the mega-alignment of Pyron and Wiens [10]. Pyron and Wiens’ [10] data set covers the entirety of the Amphibia but it seems reasonable to suppose that including sequence information for non-anuran amphibians or for some groups within Anura to which *Ericabatrachus* clearly does not belong would not be helpful. Inclusion of distantly related sequences (e.g. salamanders and caecilians) would be at a cost of increased computational complexity and would potentially lead to suboptimal model selection for the phylogenetic problem at hand. Accordingly, we restricted our attention to Ranoidea (*sensu*[6,10]), because there seems to be little doubt that *Ericabatrachus* is a member of this taxon [5,9].

The Ranoidea mega-alignment derived from Pyron and Wiens [10] was decomposed into its constituent genes. Names of samples in the alignment were preserved according to those given by Pyron and Wiens [10]. For each gene, taxa with only missing data and empty columns (alignment gaps) were deleted. For all protein-coding genes, first, second, and third codon positions were identified, and reading frames verified using Mega v.5 [26]. In the case of the non-coding *12S* and *16S* partitions, the alignments were only inspected by eye and no obvious problems were found.

New sequences for *Ericabatrachus* and for some other potentially highly relevant species that were not included in Pyron and Wiens’ [10] original work, namely the *16S* data for *Petropedeteseuskircheni*, *P. perreti*, *P. juliawurstnerae*, *P. vulpiae*, and *P. johnstoni* (retrieved from GenBank, see Additional file 4) were added to the corresponding alignments using the profile method in Muscle v.3.7 [27]. The data were further extended by the addition of *28S* sequences for all the included species for which this nuclear marker was available in GenBank (see Additional file 4) using the structure-based alignment of Mallat et al. [28] as a reference (after having deleted all non-amphibian species and having removing all gap-only columns). A final round of verification was performed during which the alignments were opened in Jalview v.2.6 [29], inspected by eye and modified as necessary, and single-gene trees were built to test for possible sequencing errors in the newly added data (by looking for unusual resolutions of the new taxon). Only low supported conflicts were observed from this analysis (reported in Additional file 5), and so the new sequences were incorporated. Ultimately, our initial concatenated, pruned and extended Ranoidea mega-alignment included the following markers (and numbers of sequences in parentheses) for a total of 858 species: *12S* (645), *16S* (795), *cytb* (244), *28S* (144), *H3A* (141), *RAG1* (258), *CXCR4* (56), *SLC8A1* (73), *POMC* (45), *RHOD* (340), *SIA* (114), *SLC8A3* (52) and *TYR* (301).

### Saturation analysis

We investigated saturation in alternative data partitions (genes and codon positions) using saturation plots generated using the program Patristic v.2 [30] from tip-to-tip distances for corresponding pairs of taxa on trees derived using uncorrected distances (p-distance) and the HKY85 + Gamma (G) model. Partitions that did not display substantial deviations from a linear regression pattern between the observed p-distances and the HKY85 distances are not saturated. In contrast, a plateau (i.e. increasing HKY85 distances correspond to non-increasing observed distance) is indicative of sequence saturation [21,23]. Saturation plots also allow the identification of sequences that are highly dissimilar from their putative homologs in the data set (probably due to poor curation or contamination). Saturated partitions and outlier sequences (with extremely high tip-to-tip distances with respect to all the other sequences in the data set) were excluded in an attempt to minimize the potential emergence of saturation-driven tree reconstruction artifacts.

### Phylogenetic analysis: a two-tiered approach

Given previous disagreement and uncertainty over the phylogenetic placement of *Ericabatrachus*, a “large-scale” approach was initially employed (including all 858 species in our Ranoidea alignment) rooted at Hemisotidae (arising from one of the basalmost splits within Ranoidea following [6,10,19]). Maximum likelihood (ML) inferences and non-parametric bootstrapping were carried out using RAxML v.7.2.6 [31]. For this analysis, unlinked GTR + GAMMA (GTR + G) models were used across the different gene and codon partitions. Additionally, we investigated the use of a partitioned model, identified using PartitionFinder v.1.0.0 [32], which suggested that some of the partitions we initially defined should be merged. The PartitionFinder model separated the data according to codon position and whether they had mitochondrial or nuclear origin. For comparison, we conducted a parallel large-scale analysis in which we used the Ranoidea section of the Pyron and Wiens [10] tree as a topological constraint, with only the positions of the newly introduced taxa (*Ericabatrachus* and some *Petropedetes*) unconstrained.

Subsequent, "small-scale" analyses were performed using a subset of taxa, selected on the basis of the large-scale ML analyses and their relative completeness, to better contextualize and further investigate the phylogenetic relationships of *Ericabatrachus*. The small-scale data set (66 taxa and 8216 bp) included *E. baleensis* and all species belonging to Petropedetidae, Pyxicephalidae (comprising Pyxicephalinae + Cacosterninae), Conrauidae and Micrixalidae. Additionally, two representatives (chosen such as to minimise missing data) from each of the Ptychadenidae, Phrynobatrachidae, Ceratobatrachidae, Dicroglossidae, Mantellidae, Ranidae and Rhacophoridae clades were included as outgroups, based on results of the large-scale analysis. Using this small-scale data set allowed missing data to be reduced (from 78% in the large scale dataset to 65% in the smaller dataset) and the use of Bayesian inference under the often better-fitting CAT-based models in PhyloBayes v.3.3 [33]. Three separate Bayesian analyses were performed with GTR + G, CAT + G, and CAT-GTR + G models. Two chains of 11230, 10900 and 22900 cycles were performed, respectively. Convergence was assessed for each analysis, with a sampling frequency of 100 and the initial 1000 trees (~10%) in each Monte Carlo Markov Chain run being discarded as burn-in. For comparison, a ML GTR + G analysis of this data set was also performed (using RAxML). In all ML analyses support values were estimated using non-parametric bootstrap (100 replicates) and all phylogenetic trees were visualized in iTOL v.2.1 [34].

Approximately Unbiased (AU) tests of two trees were used to compare the fit to the small-scale data of our new and the previously proposed (Figures 1B-E) hypotheses of the relationships of *Ericabatrachus* not including Largen's [5] very incompletely resolved hypothesis (Figure 1A). A total of eight trees was tested: those in Figures 1B to 1E, plus our Bayesian (GTR + G, CAT + G and CAT-GTR + G) trees and ML (GTR + G) tree. To compare trees in Figures 1B to 1E with our results, a preliminary series of AU tests was performed (under GTR + G) including only the trees generated from our analyses. Site-wise log-likelihoods were recalculated (for each of these topologies under GTR + G) in RAxML, and these likelihood values were used to estimate significance in CONSEL v.0.2 [35]. The tree with the best overall fit was our Bayesian GTR + G tree. This tree was then selected as the backbone to generate (by manually editing the position of *Ericabatrachus* and other taxa), trees representing the hypotheses in Figures 1B to 1E. By using the tree that provided the best fit to the data (from our preliminary AU analyses) we avoided introducing a potential bias that might have disfavored previous hypotheses not on the grounds of their placement of *Ericabatrachus* but because of the relationships they displayed for other irrelevant taxa. The trees representing the previous hypotheses and the trees from our original analyses were then subjected to another round of AU tests (under GTR + G). Additionally, we pruned the newly added taxa (*Ericabatrachus* and some *Petropedetes* species) from our Bayesian tree, used the strict consensus to compare this topology with that of the Pyron and Wiens [10] tree restricted to the common taxa, and used AU tests to compare the fit of these two trees to our and to Pyron and Wiens [10] data (under GTR + G) restricted to the subset of taxa.

### Availability of data

The datasets used for the analyses of this study are available in TreeBASE (Study Accession URL: http://purl.org/phylo/treebase/phylows/study/TB2:S15260?format=html).

New sequences produced in this work were uploaded in Genbank (accession numbers from KF938362- KF938372), more details are provided in Additional file 3. A list of sequences added to the original alignment of Pyron and Wiens [10] is provided in Additional file 4. Other additional results supporting the findings of this study can be found in Additional files 1, 2 and 5.

## Abbreviations

12S: 12SrRNA; 16S: 16SrRNA; 28S: 28SrRNA; H3A: histone 3a; RAG1: recombinase activating protein 1; cytb: cytochrome b; CXCR4: C-X-C chemokine receptor type 4; SLC8A1: solute-carrier family 8 member 1; POMC: pro-opiomelanocortin; RHOD: rhodopsin; SIA: seventh-in-absentia; SLC8A3: solute-carrier family 8 member 3; TYR: tyrosinase; HKY85: Hasegawa-Kishino-Yano 85 model; G: Gamma; ML: Maximum Likelihood; GTR: General Time Reversible model; CAT: Bayesian site-heterogeneous model; AU: Approximately Unbiased; BR: Bootstrap replicates; p: p-value; MS222: Tricaine methanesulfonate; ZNHM-AAU: Zoological Natural History Museum of Addis Ababa University, Ethiopia.

## Competing interests

The authors declare that they have no competing interests.

## Authors’ contributions

KS, SPL, DJG, MW and DP designed the study and KS and DP performed the analyses. SPL, DJG, MM, AAM, RK, FG, RdS, and SAS, participated in the collection of samples. SPL generated sequence data. KS, SPL, DJG, MW and DP wrote the paper. All authors read and approved the final manuscript.

## Supplementary Material

Additional file 1 Summary of saturation plots for all the gene partitions assessed. Click here for file

Additional file 2 **Table summarizing positions for *Ericabatrachus *** **in the bootstrap trees of the large-scale reconstruction.** Click here for file

Additional file 3 Sequences produced for this study. Click here for file

Additional file 4 **Accession numbers for the sequences added to the backbone alignment used.** Only the sequences retrieved from GenBank are shown (not the ones sequenced for this study). For a list of the sequences produced for this study see Additional file 3. Click here for file

Additional file 5 **Single gene analyses for the gene partitions that included *Ericabatrachus.*** Each tree shown is a summary of the position of *Ericabatrachus* from the majority rule extended consensus of 100 non-parametric bootstraps of a single gene partition. Click here for file
