# Supplementary figures and images for: Evolutionary relationships of the Critically Endangered frog Ericabatrachus baleensis Largen, 1991 with notes on incorporating previously unsampled taxa into large-scale phylogenetic analyses
Source: BMC Evol Biol. 2014 Mar 10;14:44. doi: 10.1186/1471-2148-14-44 (PMC4008257; doi:10.1186/1471-2148-14-44)

## Protein Coding genes

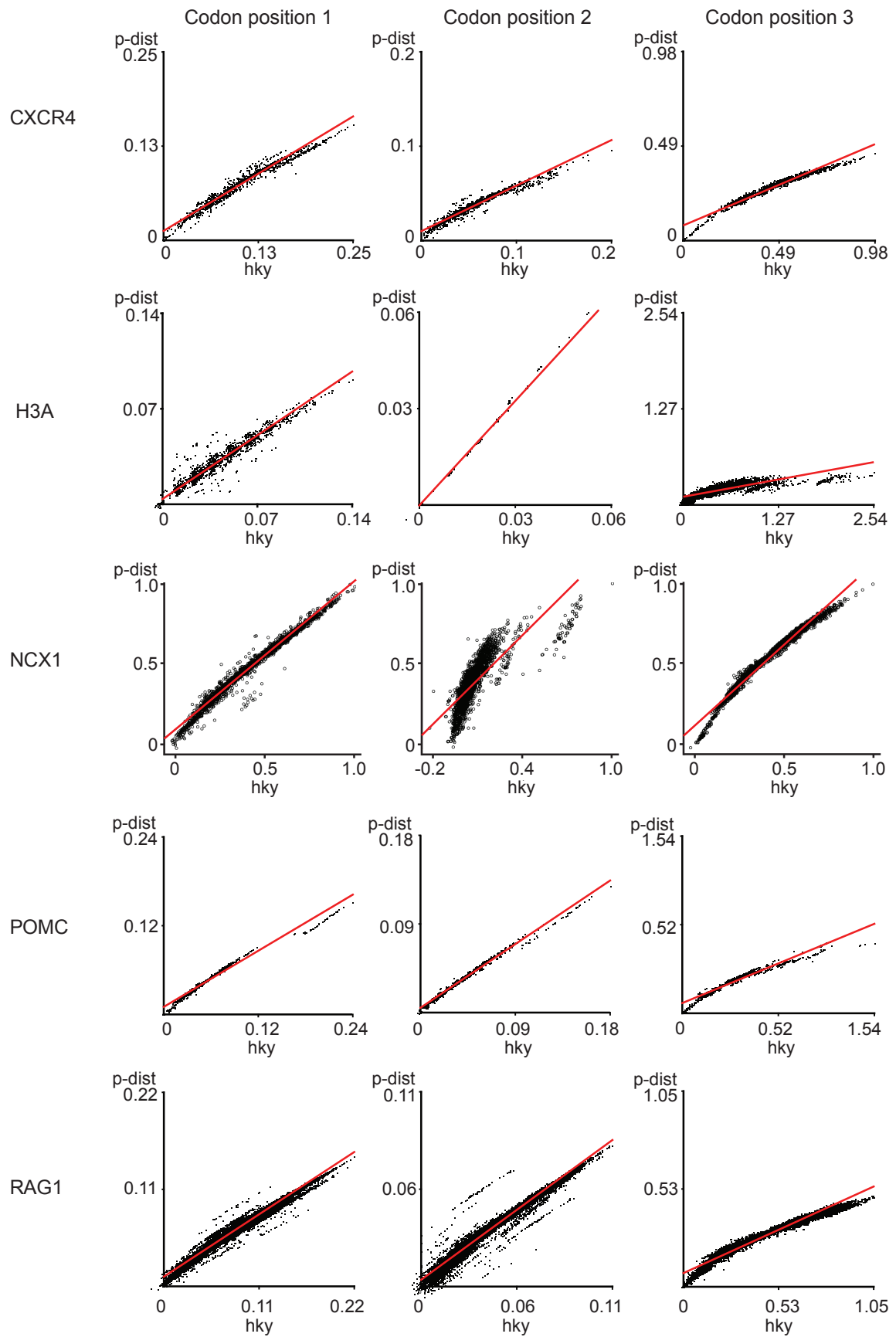

# Protein Coding genes (continued)

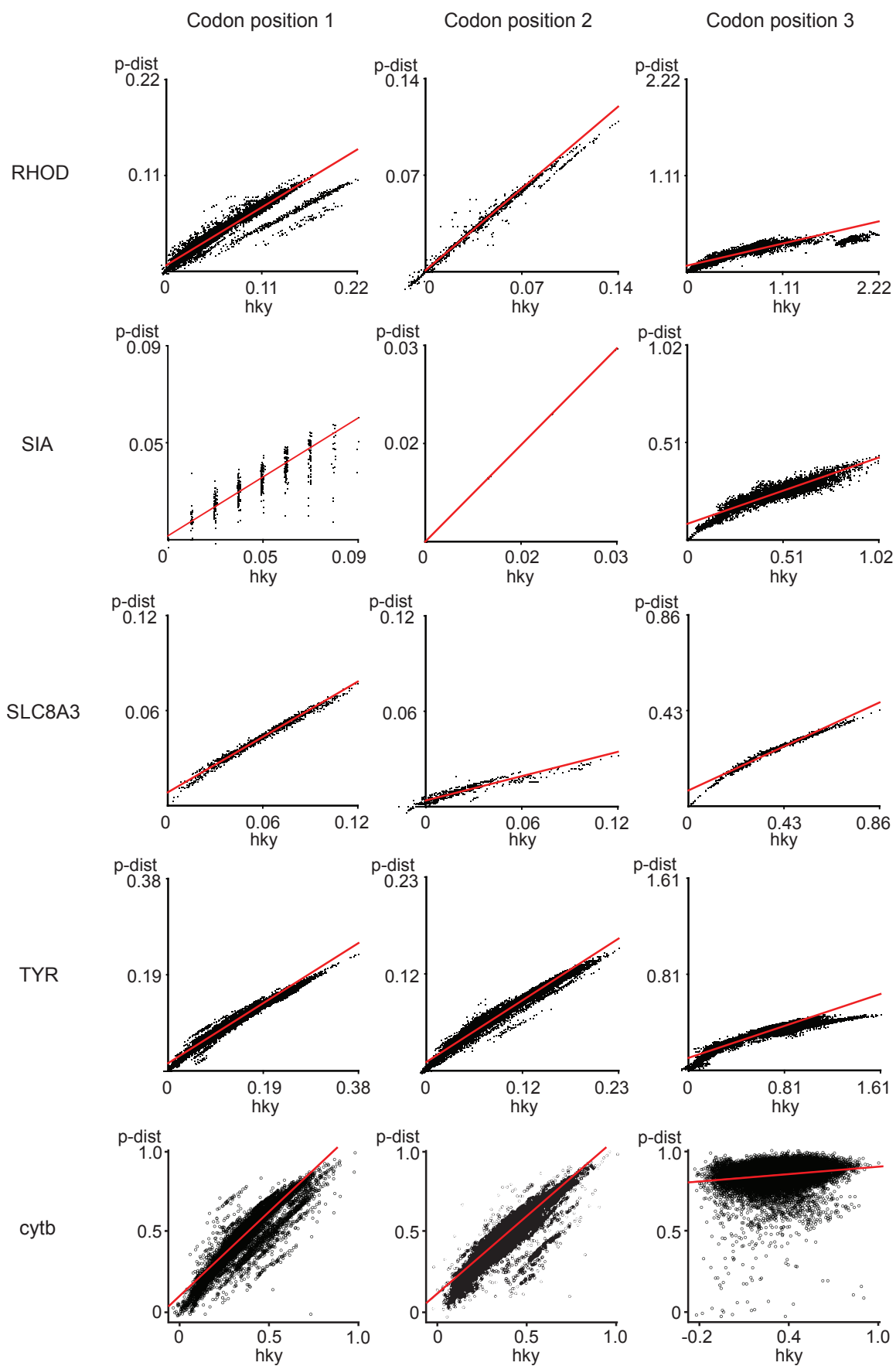

## Non Protein Coding Genes

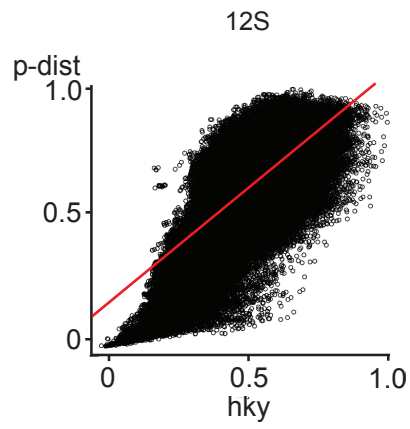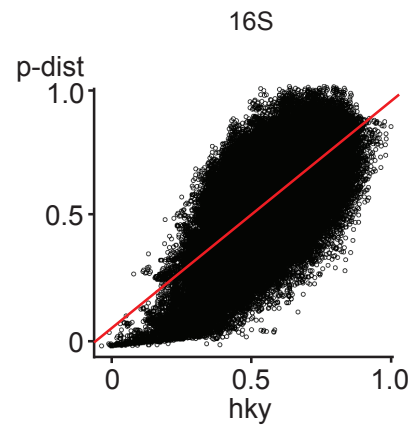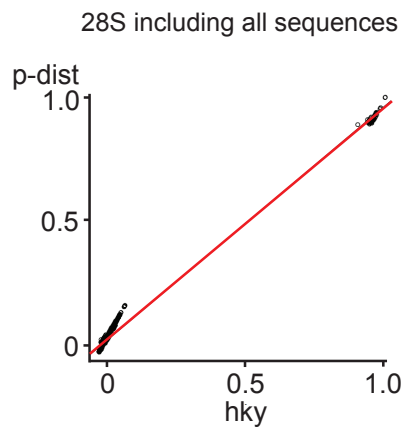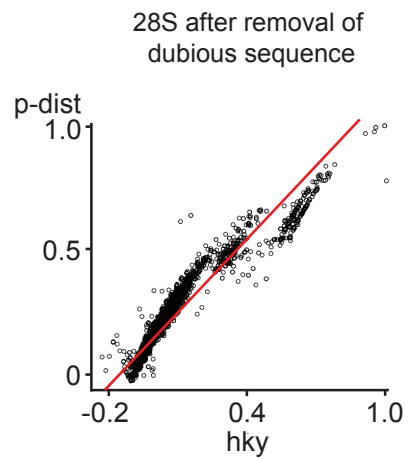

Supplement: Additional file 1 — Summary of saturation plots for all the gene partitions assessed. [file 1471-2148-14-44-S1.pdf]
