# Supplementary material for: Evolutionary relationships of the Critically Endangered frog Ericabatrachus baleensis Largen, 1991 with notes on incorporating previously unsampled taxa into large-scale phylogenetic analyses
Source: BMC Evol Biol. 2014 Mar 10;14:44. doi: 10.1186/1471-2148-14-44 (PMC4008257; doi:10.1186/1471-2148-14-44)
Supplement: Additional file 3 — Sequences produced for this study. [file 1471-2148-14-44-S3.pdf]

Additional file 3 – Sequences produced for this study.

Table S1. GenBank accession numbers for the *Ericabatrachus baleensis* sequences generated in the present study.

No./ Molecular Accession	Voucher and Field Number	Locality	12S	16S	28S	H3A	RAG1
T880	ZNHM-AAU-A2013-003 SL 065	Fute, Harena Forest, Bale Mountains	KF938362	KF938365	KF938368	KF938369	KF938370
T1083	ZNHM-AAU-A2013-001 AK 2020	Fute, Harena Forest, Bale Mountains	KF938363	KF938366	-	-	KF938371
T1084	ZNHM-AAU-A2013-002 AK 2022	Fute, Harena Forest, Bale Mountains	KF938364	KF938367	-	-	KF938372

Table S2. Primers used in this study.

Refer to text for abbreviations.

Gene	Primer
12S	12S A-L: AAACTGGGATTAGATACCCCACTAT<br>12S F-H: CTTGGCTCGTAGTTCCCTGGCG
16S	16AR: CGCCTGTTTATCAAAAACAT<br>16Br: CCGGTCTGAACTCAGATCACGT
RAG1	RAG 1c: GGAGATGTTAGTGAGAARCAYGG<br>RAG 1e: TCCGCTGCATTTCCRATGTCRCA
28S	28Sv: AAGGTAGCCAAATGCCTCATC<br>28Sjj: AGTAGGGTAAAACTAACCT
H3A	h3F: ATGGCTCGTACCAAGCAGACVGC<br>h3R: TATCCTTRGGCATRATRGTGAC

Note on intraspecific variation

The three samples of *Ericabatrachus baleensis* presented no variation at the nuclear RAG1 and mitochondrial 12S and 16S.
