# Supplementary material for: Evolutionary relationships of the Critically Endangered frog Ericabatrachus baleensis Largen, 1991 with notes on incorporating previously unsampled taxa into large-scale phylogenetic analyses
Source: BMC Evol Biol. 2014 Mar 10;14:44. doi: 10.1186/1471-2148-14-44 (PMC4008257; doi:10.1186/1471-2148-14-44)
Supplement: Additional file 5 — Single gene analyses for the gene partitions that included Ericabatrachus. Each tree shown is a summary of the position of Ericabatrachus from the majority rule extended consensus of 100 non-parametric bootstraps of a single gene partition. [file 1471-2148-14-44-S5.pdf]

Single gene analyses

Please note the names for taxa here and in all trees presented in this study are given according to those downloaded from Genbank. Therefore some taxonomic names are incorrect, e.g. *Petropedetes newtoni* is a synyonym of *P. johnstoni* following [1].

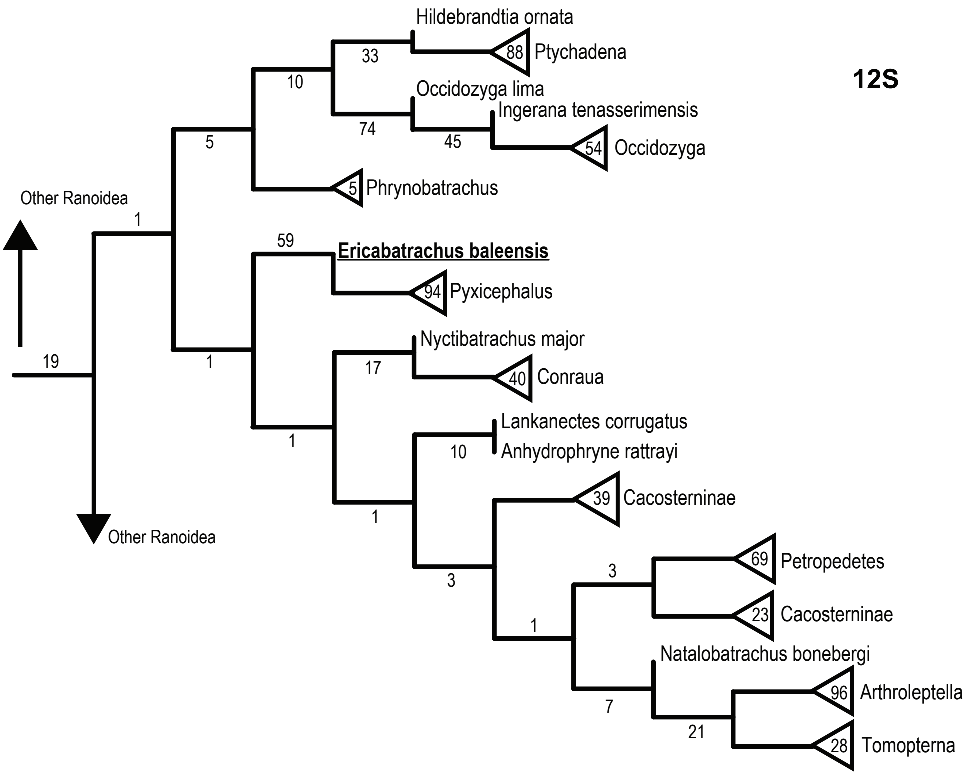

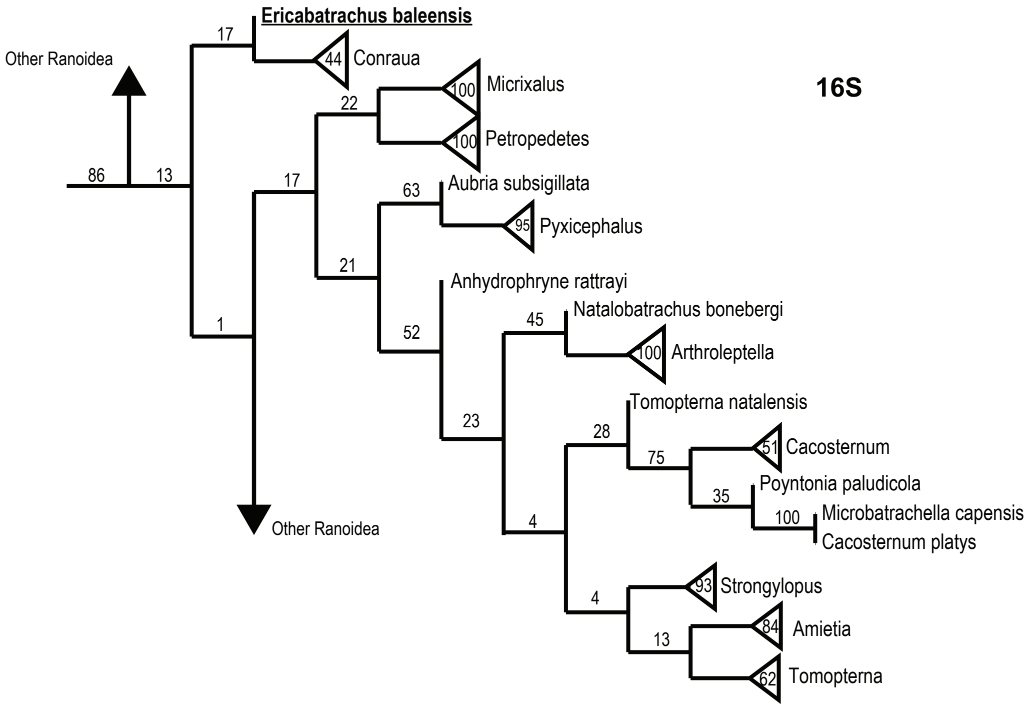

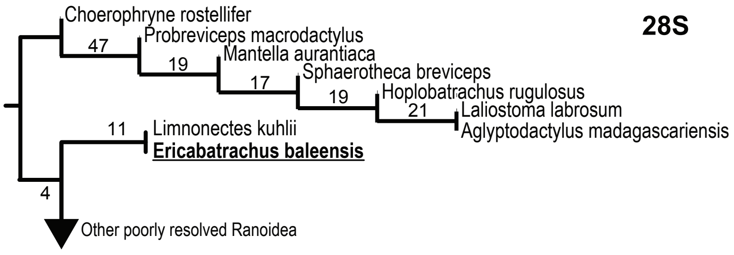

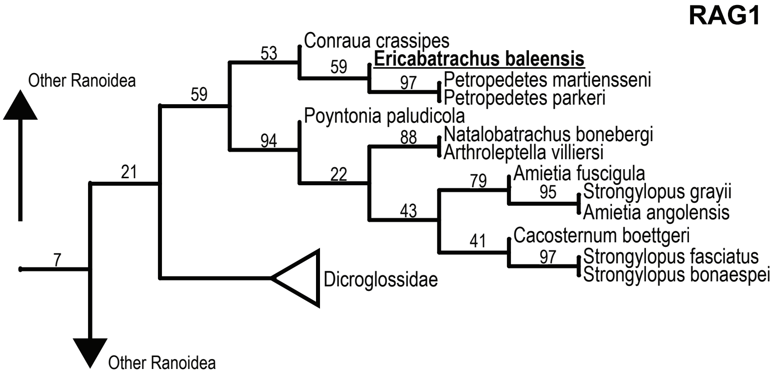

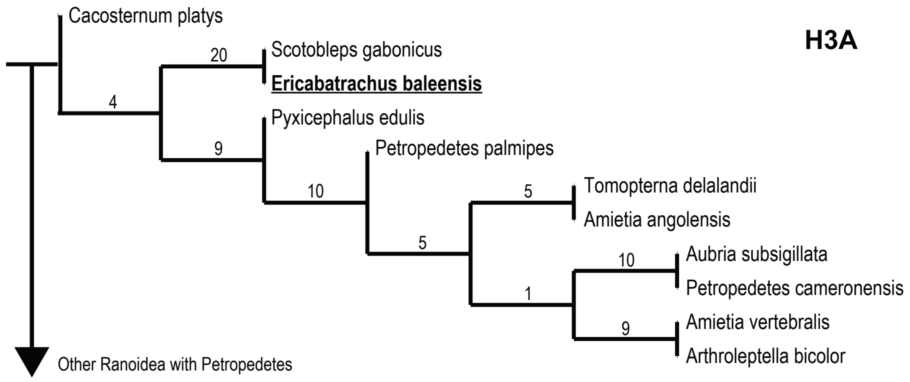

Reference

Barej MF, Rödel M-O, Gonwouo LN, Pauwels OSG, Böhme W, Schmitz A: **Review of the genus *Petropedetes* Reichenow, 1874 in Central Africa with the description of three new species (Amphibia: Anura: Petropedetidae).** *Zootaxa* 2010, **2340:**1-49.
